# Financing of Trauma and Burn Prevention and Care in Malawi: A Scoping Review

**DOI:** 10.1002/hsr2.72462

**Published:** 2026-04-28

**Authors:** Kafayat Aminu, Oluwatobi Emmanuel Adegbile, Olubukola Omobowale, Yovanthi Anurangi Jayasinghe, Adetayo Olorunlana, Precious Chika Nnannah, Emeka Okeke, Ugochukwu Anthony Eze, Afeez Abolarinwa Salami, Rita Amarachi Nwebo, Kehinde Kazeem Kanmodi

**Affiliations:** ^1^ Centre for Evidence Synthesis and Implementation Research Cephas Health Research Initiative Inc Ibadan Nigeria; ^2^ Centre for Disease Control and Prevention Cephas Health Research Initiative Inc Ibadan Nigeria; ^3^ Department of Community Medicine University of Ibadan Ibadan Nigeria; ^4^ Department of Research and Innovation University of Technology and Entrepreneurship Phnom Penh Cambodia; ^5^ Scientific Advisory Board Cephas Health Research Initiative Inc Ibadan Nigeria; ^6^ Centre for Digital Health Research, Innovation and Practice Cephas Health Research Initiative Inc Ibadan Nigeria; ^7^ Office of the Executive Director Cephas Health Research Initiative Inc Ibadan Nigeria; ^8^ Centre for Dental and Craniofacial Research Cephas Health Research Initiative Inc Ibadan Nigeria; ^9^ School of Health and Life Sciences Teesside University Middlesbrough UK; ^10^ Department of Public Health Thomas Adewumi University Oko‐Irese Nigeria

**Keywords:** burn injuries, cost analysis, health financing, injury prevention, Malawi, trauma care

## Abstract

**Background and Aims:**

Trauma and burns constitute major public health concerns in Malawi, disproportionately affecting children, young adults, and low‐income populations. Despite free care policies at public facilities, the financial implications of trauma and burn care are profound and understudied. This scoping review examined the evidence on financing of trauma and burn prevention and care in Malawi.

**Methods:**

This scoping review was conducted following Arksey and O'Malley's framework and reported using the PRISMA‐ScR guidelines. A systematic search of ten bibliographic databases, Google Scholar and Google was conducted up to April 2025. Relevant literature were included in the scoping review if they investigated the financing of trauma or burns prevention and care in Malawi. Included peer‐reviewed literature were appraised using the Mixed Methods Appraisal Tool (MMAT, 2018 version), while grey literature was appraised using the AACODS checklist. Data were extracted, charted, and synthesized thematically.

**Results:**

Of the 16 included literature (6 peer‐reviewed, 10 grey literature), all peer‐reviewed studies used quantitative methods, mostly descriptive. Four focused on inpatient cost analyses. The mean burn admission cost was US$559.85; and orthopaedic treatment ranged from US$195–$711. Personnel (up to 48%), medications, and facility resources were the main cost drivers. Indirect costs included transportation (US$2.87 per visit), income loss, and food insecurity. Annual trauma registry maintenance cost US$33,361.64. All the peer‐reviewed literature reported exclusive government funding with no donor or private sector financing. Grey literature confirmed no dedicated trauma/burn financing lines, relying instead on general health allocations and non‐financial support like training and donated supplies.

## 1 Introduction

Burn and trauma injuries represent a significant public health challenge in Malawi, contributing substantially to morbidity, mortality, and economic burden [1, 2, 3]. Burns account for approximately 4.4% of all injuries presenting to hospitals in Malawi, with pediatric populations disproportionately affected, around 67.6% of burn patients are under 15 years old [4, 5, 6]. Scald burns are the most common mechanism, responsible for over half of these injuries, and the average burn surface area among admitted patients is about 14% [6, 7, 8]. Hospital mortality rates for burn patients remain alarmingly high, with figures reaching up to 27%, and wound infection rates around 31% [1, 2, 3, 6]. Between August 2018 and June 2021, trauma registries at 10 major hospitals recorded over 118,000 trauma cases, with young males and adults disproportionately affected [9]. Road traffic crashes (RTCs) account for nearly half (48%) of all admitted trauma cases, and falls represent 46% of all trauma presentations [9]. The demographic most impacted includes farmers, students, and manual laborers, reflecting the vulnerability of Malawi's working‐age population and stressing the critical gaps in care delivery [9].

The financial burden of burn and trauma care in Malawi is profound, impacting both healthcare facilities and patients' families [10, 11]. Burn care requires prolonged hospital stays averaging 21 days, specialized interventions such as debridement and amputation, and intensive resource use in under‐resourced settings, which strains the already limited health infrastructure [6, 12]. While the incidence of trauma injuries is projected to rise, with road traffic injuries alone expected to double by 2030 if current trends persist, further straining the healthcare system [13]. Given Malawi's status as the fourth poorest country in sub‐Saharan Africa, with a predominantly rural population and constrained healthcare funding, the economic implications are severe [14, 15]. Although precise national expenditure data on burn and trauma care are scarce, applying global health economic valuation methods suggests substantial costs [16, 17]. Notably, health system financing deals with the overall funding, pooling, and purchasing of services for the entire population across all types of care, whereas trauma‐specific financing is a targeted funding mechanism for high readiness costs and specialized, immediate care for injured patients [18, 19]. In 1997, the U.S. Environmental Protection Agency estimates the Value of a Statistical Life at approximately US$5.8 million [20]. Adjusting this figure to Malawi's demographic and economic context in 2025 indicates that each preventable death from burns or trauma represents a significant economic burden [20, 21, 22].

Recent multi‐site data collection efforts in Malawi, highlighted the extensive scale of injury burden and the urgent need for effective financing strategies to support prevention and control programs [9, 23]. Despite this, there remains a paucity of comprehensive data on the financing of burn and trauma prevention and control in Malawi, limiting the ability to design sustainable interventions and allocate resources efficiently. This scoping review aims to examine the existing literature on the funding sources, the direct and indirect costs, and challenges of financing of burn and trauma prevention and care in Malawi. By mapping current financing strategies and identifying gaps, this review seeks to inform policymakers and stakeholders on optimizing resource allocation to reduce the burden of burns and trauma in Malawi.

## 2 Methods

### 2.1 Title and Protocol Registration

The title and protocol of this scoping review were registered in the Open Science Framework registry (https://osf.io/yw2jp).

### 2.2 Review Design

We leveraged on the step‐by‐step recommendations for designing scoping reviews that were proposed by Arksey and O'Malley to guide the documentation and conduct of this scoping review [24]. Also, the Preferred Reporting Items for Systematic Reviews and Meta‐analyses extension for Scoping Reviews (PRISMA‐ScR) guidelines were used to report the findings of this review [25].

### 2.3 Research Question Identification

This scoping review sought to answer the research question: How are trauma and burn prevention and control financed in Malawi? To extensively map the existing evidence concerning this research question, the question is broken down to the following sub‐questions:

i. What funding sources exist for the prevention and care of trauma and burn in Malawi?
ii. What are the direct and indirect costs of the prevention and care of trauma and burn in Malawi?
iii. What are the challenges associated with the financing of trauma and burn prevention and care in Malawi?

### 2.4 Literature Search

The research team developed a robust search strategy based on the objectives of this review. The search strategy was efficiently designed to proactively identify and retrieve studies that potentially align with the goals of this review. Members of the research team made several inputs to the search strategy, after which a unified strategy was agreed upon through consensus for the purpose of our literature search. For the financing subject terms, the following terms were utilized: “donat*” “fund*” or “invest*” or “money” or “bank*” or “grant*” or “loan” or “budget*” or “allocat*” or “donor” or “sponsor*” or “aid” or “financ*.” For the trauma and burn subject search, the following terms were used: “trauma*” OR “accident*” OR “injur*” OR “fracture*” OR “wound” OR “burn” OR “scald.”

We carried out a systematic search on April 1, 2025, to identify and retrieve all relevant studies published from inception till date from PubMed, SCOPUS, and AMED (These include the Allied and Complementary Medicine Database), CINAHL Ultimate, Dentistry and Oral Sciences Source, SPORTDiscus with Full Text, APA PsycArticles, Psychology and Behavioral Sciences Collection, and APA PsycInfo.

In addition, gray literature was identified through structured searches of Google Scholar and Google to capture relevant policy documents, program reports, and government financial and budgetary records related to the financing of trauma and burn prevention and care in Malawi.

Tables S1–S4 (Supporting File) present the search strings used for the bibliographic database and gray literature searches.

### 2.5 Eligibility Criteria

For this review, only those studies that met the following criteria were included:

- Empirical studies published in peer‐reviewed journals
- Quantitative, qualitative, and mixed methods studies
- Empirical studies focused on the financing of trauma and burn prevention and care
- Empirical studies conducted in Malawi or that utilized Malawi as their country of context
- Studies with accessibility to full texts
- Studies published in English
- Studies published from inception to April 2025.

For peer‐reviewed literature, the following were excluded:

- Case series, case reports, systematic reviews, scoping reviews, meta‐analyses, and letters to the editor
- Empirical studies focusing on health financing outside Malawi
- Empirical studies examining the financing of non‐trauma or non‐burn health conditions in Malawi.

### 2.6 Study Selection Process

All records retrieved from the bibliographic databases were imported into the Rayyan software for deduplication and screening. After duplicate records were removed, two reviewers (RAN and PCN) independently screened the titles and abstracts of peer‐reviewed studies using the predefined eligibility criteria, and any disagreements at this stage were resolved by a third reviewer (OEA).

The studies that passed this initial screening were then assessed in full text by RAN and PCN, with differences in judgment resolved through discussion and, where necessary, with input from OEA. The final list of included and excluded peer‐reviewed studies resulting from this process is presented in Table S5a (Supporting File).

In parallel, gray literature identified through searches of Google Scholar and Google was screened separately for relevance to the review objectives, and the documents that met the inclusion criteria following this process are presented in Table S5b (Supporting File).

**Quality Appraisal**

The quality of the included peer‐reviewed studies was assessed using the Mixed Methods Appraisal Tool (MMAT), 2018 version [26]. The MMAT provides a structured approach for appraising qualitative, quantitative, and mixed‐methods studies through two initial screening questions, followed by five design‐specific criteria tailored to the methodological approach of each study. Each criterion was rated as *“Yes,” “Can't tell,”* or *“No,”* enabling a transparent assessment of methodological strengths and limitations.

Gray literature sources (*n* = 10) were appraised using the AACODS checklist, developed by Tyndall (2010), which is specifically designed for the assessment of non‐peer‐reviewed evidence such as government reports, policy documents, and organizational publications. The AACODS framework evaluates sources across six domains: Authority, Accuracy, Coverage, Objectivity, Date, and Significance, allowing for a systematic appraisal of credibility, transparency, and relevance. Appraisal judgments were recorded as *“Yes,” “No,” “Not applicable,”* or *“Not stated.”*

All included peer‐reviewed studies and gray literature sources were independently appraised by two reviewers (YAJ and EBO). Any discrepancies were resolved through discussion, with consultation of a third reviewer (KKK) where necessary to ensure consistency in methodological assessment. The outcomes of the quality appraisal are presented in Tables S6–S10 (Supporting File). Consistent with scoping review methodology, no studies were excluded on the basis of quality appraisal; rather, appraisal findings were used to support contextual interpretation of the results.

### 2.7 Data Charting

The lead author (KA) developed the preliminary version of the data extraction template, and it was reviewed by three authors (RAN, PCN, and KKK). After several revisions and inputs among these team members, a final template was approved to extract key information that critically addresses the objectives of this review. The lead author (KA) independently carried out the data extraction procedure using a Microsoft Excel spreadsheet to organize and collate all information. Three authors (RAN, PCN, and KKK) reviewed the extracted data and ensured that all information was comprehensively and accurately extracted. In case there were conflicts, consensus was reached on all issues. The following information was extracted from all included studies: study title, lead study author, study location/region in Malawi, study design, study sample size, study aims or objectives, study focus (trauma or burns), type or severity of trauma, sample characteristics (age, sex, race or ethnicity), participant recruitment method (household survey, hospital survey, clinic survey, death records or certificates etc.), review objectives, source or type of trauma or burns prevention and control financing (e.g., not‐for‐profit organizations, academic institutions, government [federal, state, or local], individual, faith‐based enterprises, research organizations or ventures, and other sources), funding amount, funding timeline or duration [rolling/recurrent/or one‐time], funding identifier, study outcomes (primary and secondary), and other relevant information.

### 2.8 Collation and Presentation of Data Charting

The extracted data were collated and summarized using a narrative synthesis approach [27]. In the synthesis procedure, codes were generated from the extracted data, after which these codes were grouped into themes by the lead author (KA) and shared with three authors (RAN, PCN, and KKK) to review. These themes were reviewed back‐and‐forth by these three authors till an accurate and comprehensive data summarization was accomplished. The summarized data were presented in texts, tables, and charts.

## 3 Results

The database search yielded 288 records across seven bibliographic databases, namely PubMed (*n* = 83), SCOPUS (*n* = 163), AMED, the Allied and Complementary Medicine Database (*n* = 3), APA PsycINFO (*n* = 7), CINAHL Ultimate (*n* = 27), Regional Business News (*n* = 2), and the Psychology and Behavioral Sciences Collection (*n* = 3). All records were imported into the Rayyan software, where 119 duplicates were identified and removed.

After deduplication, 169 unique records remained and were screened based on their titles and abstracts. During this stage, 155 records were excluded because they did not meet the eligibility criteria. This process resulted in 14 articles being selected for full‐text review. Following full‐text assessment, eight articles were excluded due to reasons such as irrelevant objectives, inappropriate study design, unsuitable outcomes, or publication type. In total, six peer‐reviewed studies met the inclusion criteria and were included in this scoping review. A detailed summary of the included and excluded peer‐reviewed studies is provided in Table S5a.

In addition to the database search, a gray literature search was conducted using Google Scholar. This search identified 14 potentially relevant documents. After full‐text screening, four documents were excluded, with two excluded due to wrong study objectives and two excluded due to wrong study outcomes. Consequently, 10 gray literature documents met the inclusion criteria and were included in the review. These comprised policy reports, program descriptions, and government and donor financial records that provided contextual information on the financing of trauma and burn prevention and care in Malawi. The gray literature sources included in the review are presented in Table S5b.

**Figure 1:**
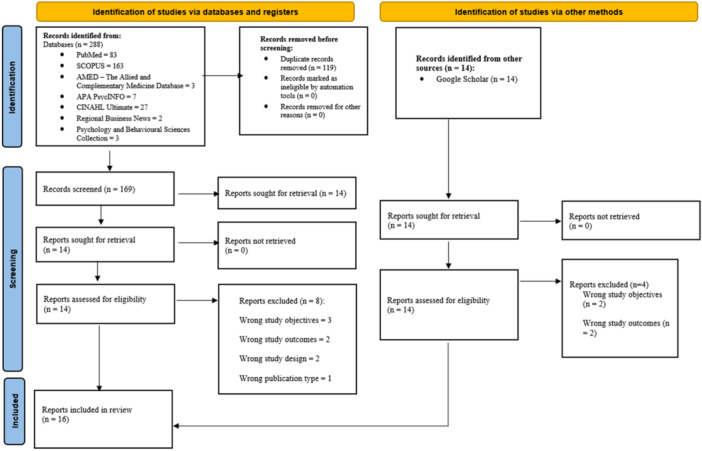
Flow chart depicting the literature identification, deduplication, screening, and selection processes.

The full study selection process for both peer‐reviewed and gray literature is illustrated in Figure 1.

### 3.1 Study Focus

This scoping review maps the existing evidence on the financing of trauma and burn prevention and care in Malawi, a low‐resource setting, drawing on both peer‐reviewed studies and gray literature. Among the six peer‐reviewed studies included, the majority focused on the economic costs and financial consequences of trauma and burn care, while a smaller number explored system‐level barriers affecting access to care. Likewise, the focus of the 10 gray literature vary, and they were on national economic development and planning, financial and macro‐economic analysis, and health matters.

Specifically, four studies (66.7%) examined costs and economic burden associated with trauma and burn care, including hospital‐level cost analyses, patient‐level socioeconomic impacts, and health system expenditure related to injury and orthopedic services [28–31]. These studies provided evidence on direct medical costs, resource utilization, and the broader economic implications of trauma and burn care within Malawi's health system. The remaining two studies (33.3%) focused on barriers to effective trauma care delivery, including access to quality injury care and system‐level constraints affecting trauma services [11,32]. As for the gray literature, four addressed economic development analysis, budgeting, and fiscal planning [28, 29, 30, 31], while one focused on water‐related climate shocks [32]. Five reported healthcare‐related issues, including activities within the general healthcare sector [33] and emergency trauma and burn care [34, 35, 36, 37].

#### 3.1.1 Study Design/Document Type

The included peer‐reviewed studies employed study designs that were appropriate to their research objectives and the Malawian context (Table 1). Two studies by Whitaker et al. [11,32] adopted cross‐sectional descriptive designs to assess access to injury care and barriers within health facilities. Gallaher et al. [28], Purcell et al. [30], and Twea et al. [31] employed quantitative descriptive designs to estimate the costs and resource requirements associated with burn, trauma, and orthopedic care. In contrast, Mody et al. [29] used a cross‐sectional analytical design to examine the socioeconomic consequences of femoral shaft fractures among patients in Malawi.

**Table 1:** Summary of studies on trauma and burn financing in Malawi.

S/N	Authors & date	Study design	Objective	Methodology	Study population	Age distribution	Sample Size	Type of trauma investigated	Study timeline	Major findings	Limitations	Conclusion & recommendations
1.	Whitaker et al., 2024 [11]	Cross‐sectional descriptive study	To assess injury incidence, health‐seeking behavior, time delays, and barriers to care.	Household survey in 2200 households; injury and care utilization data collected	Community‐based population in Karonga HDSS	Median age at injury: 22 (IQR 10–40); 61.9% male	531 households (611 non‐fatal and 4 fatal injuries)	Fatal and non‐fatal injuries	12 months (October 2019– February 2020)	Barriers to seeking care for injuries were associated with financial cost of care and transportation	Self‐reported data, recall bias, limited generalizability beyond HDSS	The injury burden is substantial; need to address barriers to prompt care and improve facility capabilities
2.	Gallaher et al., 2015 [38]	Quantitative descriptive study	To estimate the costs of daily inpatient burn care at a tertiary burn center in Malawi	Activity‐based costing for all admitted patients, a bottom‐up cost analysis methodology	All patients admitted to KCH Burn Unit (June 2011–Aug 2014)	Median age: 3 years (IQR 2–10); 80% <18 years; 55% male	905	Burns	3 years (June 2011 and August 2014)	Mean daily per‐patient cost: US$24.26; mean cost per admission: US$559.85; mean daily cost per 1% TBSA: US$2.65; facility and medication costs were largest contributors	Excluded outpatient costs; building depreciation estimate unavailable; regional cost variability; retrospective design	Comprehensive burn care is affordable and sustainable in a low‐income setting with moderate funding; accurate cost data is essential for policy and planning
3.	Whitaker et al., 2024 [39]	Cross‐sectional descriptive study	To identify and rank perceived barriers to injury care using the Three Delays framework (seeking, reaching, and receiving)	Survey of 228 staff across 11 facilities	Health facility staff involved in injury care	Not specified (staff demographics only)	228 staff (female‐84, male‐144)	Trauma	3 months (22nd July and 30th October 2019)	Delay 3 (receiving care) is most perceived as critical. Perceived barriers include lack of physical resources, financial costs of care, transport cost, and staff	Based on staff perception, not patient data; potential bias	Investment in infrastructure, staff training, and emergency transport is essential to improve injury care delivery
4.	Purcell et al., 2020 [40]	Quantitative descriptive study	To analyze the cost of creating and maintaining a hospital‐based trauma registry in Malawi	Retrospective analysis of prospectively collected data (2018–2019). Activity‐based costing (bottom‐up cost analysis) training	All trauma patients presenting to KCH casualty from scene or transfers, including dead‐on‐arrival cases	Not specified	12,616	Trauma	1 year (2018–2019)	− Startup cost: US$3,196.24 (personnel, database, training, supplies). − Annual cost: US$33,361.64 (personnel, technology, supplies, facility). − Cost per patient registered: US$2.64 in 2018.	− Data limited to hospital‐based cases; may miss pre‐hospital deaths or cases not presenting to KCH. − Generalizability may be limited to similar resource‐poor settings	Anticipating the financial burden is imperative for sustainability. Recommend continued investment in personnel, training, and infrastructure for effective trauma surveillance and quality improvement.
5.	Twea et al., 2024 [41]	Quantitative descriptive Study	To estimate the economic costs of orthopedic services at tertiary hospitals in Malawi	‐ Mixed costing methodology: Top‐Down (hospital‐level costs) and Time‐Driven Activity‐Based Costing (TDABC) for diagnosis‐specific costs.	All patients receiving orthopedic services during the study period	Not explicitly stated; majority adults, but includes all ages	2,372 patient files reviewer (72% male)	Orthopedic injuries	Not specified	− Mean weighted cost of treatment ranged from US$195 (supracondylar fractures) to US$711 (proximal ulna fractures). − Main cost components: personnel (30%), medicines & supplies (23%). − Length of hospital stay was the most significant cost driver, explaining disparities between hospitals. − Substantial variation in costs and resource utilization between hospitals. − Orthopedic care is a major health system expenditure in Malawi.	− Indirect patient costs (out‐of‐pocket or societal costs) not included. − Only two hospitals studied, hindering generalizability. − Diagnosis‐specific costs limited to most prevalent conditions.	− Orthopedic care is critical and resource‐intensive in LMICs, with significant variation in cost drivers and resource use. − Recommend future research on cost‐effectiveness of specific orthopedic interventions, and expansion to other hospitals and broader patient costs.
6.	Mody et al., 2023 [42]	Cross‐sectional analytical study	To examine the socioeconomic consequences of femoral shaft fractures for patients in Malawi.	‐ Sub‐study of a larger prospective cohort (original *n* = 187)	Adults with femoral shaft fractures treated at KCH and QECH (both operative and non‐operative cases)	Mean age: 36.0 ± 14.8 years; 81% male, 19% female	42 (completed socioeconomic survey)	Traumatic femoral shaft fractures	1 year	− 29% reported decreased household income. − 49% reported household food insecurity in the week prior to survey. − Many reported selling property, borrowing money, changing residence, or unenrolling children from school due to financial hardship. − Average transport cost per hospital visit: US$2.87 (0.5% of GDP per capita). − 76% required unpaid caregivers (average 11.9 weeks off work).	− Small sample size for socioeconomic survey (*n* = 42). − Only two hospitals included. − Did not capture all indirect costs or long‐term outcomes. − Possible recall bias in self‐reported data.	The indirect costs and socioeconomic impact of femoral shaft fractures in Malawi are substantial, affecting work, income, food security, and family well‐being. While care is free at the point of service, there is inadequate financial risk protection. Recommendation: Increase investment in trauma care capacity, prevention, and social protection.

However, the documents identified in the gray literature can be classified into 4 types: government financial/economic documents [28, 29], national reports, policy guidance and strategy documents [31, 32, 35], media reports [36], and research evidence that adopted an observational design [34] (Table 2).

**Table 2:** Summary of gray literature on trauma and burn financing in Malawi.

S/N	Authors & date	Document type/design	Objective	Methodology	Study population	Age distribution	Sample size	Type of trauma investigated	Study timeline	Major findings
1.	Interburns (2019) [36]	News or Media Report	Describes past and ongoing activities of Interburns	Not stated, describes program activities	Trainees (nurses) not research subjects	Not indicated	Not specified; Mentions “several nurses” and “2 nurses”	Focuses on burn care training	Describes ongoing training support (e.g., 2016 and 2018 courses)	Limited but targeted capacity‐building in burn care in Malawi. Many nurses participated in Advanced Burn Care (ABC) training in Ghana and Ethiopia in 2016 and 2018 Two of the trained nurses later delivered Essential Burn Care (EBC) and ABC training
2.	International Monetary Fund (2017) [31]	Country Report	To highlight the development areas that Malawi will emphasize over the planning period in order to promote her development	Policy analysis and consultation using existing data and country planning documents	No human subject involved	Not applicable	Not applicable	No trauma mentioned, health is mentioned as a development/planning priority	Years preceding development strategy period (MGDS II) and future planning (MGDS III)	Malawi experienced disappointing implementation of its previous development strategy (MGDS II), leading to a rethink of planning frameworks
3.	World Health Organization (2008) [35]	Policy/Strategic Guidance Document	To outline a comprehensive global strategy for burn prevention and care, raise awareness of the burden of burn injuries, and propose actions to reduce burn incidence and improve burn care systems worldwide.	Expert consultation	Not applicable	Not applicable	Not applicable	Burn injuries	Not indicated	Burn prevention and care are cost‐effective with the right policies and programs. Advocacy, multisectoral collaboration, and tailored prevention strategies are recommended to reduce burn incidence and improve outcomes.
4.	Republic of Malawi, Ministry of Finance and Economic Affairs (2023) [32]	Environmental and Social Management Framework (ESMF)	To improve the resilience to water‐related climate shocks in Malawi and in the Eastern and Southern Africa region.	Literature review and stakeholder consultations	Not applicable	Not applicable	Not applicable	No mention of specific trauma	Not specified	Potential environmental and social risks from climate shocks (e.g., floods, cyclones) and infrastructure works identified; Mitigation and monitoring measures indicated. No health financing or trauma care recommendations provided.
5.	Republic of Malawi, Ministry of Finance and Economic Affairs (2025) [28]	Government Budget Policy Document	To outline the national government's revenue estimates, expenditure priorities, and policy directions for the 2025/26 fiscal year	Economic forecasts, revenue projections, and stakeholder consultations	Not applicable	Not applicable	Not applicable	Not specified	Fiscal year 2025/2026	The Health and Population Sector gets K741.05 billion, about 9.2% of the national budget, to support infrastructure (hospitals, health posts), essential services, and an emergency project to sustain health services. These allocations support expansion and continuity of health services, but trauma‐specific or burn care financing are not specified within the public budget.
6.	Republic of Malawi, Ministry of Finance and Economic Affairs (2025) [29]	Government Financial Statement	To present the draft financial framework for the 2025/26 fiscal year	Fiscal planning and budget analysis, using past expenditures, economic forecasts, government policy, and statutory budget preparation processes	Not applicable	Not applicable	Not applicable	Not specified	2025/26 Fiscal Year	Health Sector Allocations (recurrent): K23,017 million for 2025/26 (compared to K18,867 million in 2024/25). Financial allocation for trauma or burn prevention and care not specified
7.	Republic of Malawi, Ministry of Finance and Economic Affairs (2026)	Macro‐economic Review of Malawi's performance (GDP, inflation, sector contributions, strategic sectors)	To provide an overview and analysis of Malawi's economy in the year under review and projections	Economic and statistical analysis using government macroeconomic data, sector performance indicators, and stakeholder and sectorial reports	Not applicable	Not applicable	Not applicable	Not specified	Fiscal year 2024/25; projections extended into 2025/26 for long‐term development strategies	Improvements recorded in service delivery, such as integrated care pathways (maternal/child health, chronic care)Gaps remain in funding, resource allocation, and workforce capacityDirect facility financing (DFF) piloted to enhance strategic purchasing of health servicesNo specific reference to burn or trauma care financing
8.	Trent‐Gurbuz (2018) [37]	Program Report	Reports activities of Africa Burn Relief Program in Malawi	Describes activities such as fundraising, donating supplies, clinical protocol development, training, and ongoing remote mentoring	Burn patients and local clinicians in Malawi served by the program	Not specified	Not specified	Burns	Reports activities from early 2000s to 2018 including volunteer, program work and ongoing missions and training.	Africa Burn Relief Program provides donated supplies, Develops treatment protocols for second‐ and third‐degree burns; Trains local clinical officers and supports remote mentoring via WhatsApp. The cost of patient care are covered because some patients often leave treatment due to inability to pay No budget allocation or formal health financing mechanisms reported
9.	The World Bank (2021) [34]	Observational Research Study	To assess the incidence and characteristics of trauma cases in Malawi, understand causes and outcomes of trauma, and evaluate early implementation services and trauma registry feasibility	Mixed‐methods	Trauma patients presenting to selected hospitals in Malawi	Not specified	118,013+ trauma cases recorded in the registry dataset across 10 facilities	Multiple trauma types, including road traffic crash (RTC) injuries, falls, and other trauma	August 2018 ‐ June 2021	Gaps identified in formal emergency medical systems such as pre‐hospital care and coordinated response. No information on health financing for trauma and burn, specific budget allocations dedicated for trauma and burn not revealed
10.	World Health Organization (2025) [33]	Health System Report and Descriptive Summary of WHO Support	To present progress, priorities, and achievements in Malawi's health sector over 2024	Collation of data from health system performance, program outcomes, emergency responses, and WHO support activities	Not applicable	Not applicable	Not applicable	Not specified	Report covers WHO support and national health activities during 2024	Report emphasizes strengthening primary health care and emergency preparedness National Health Accounts and Direct Facility Financing were institutionalized in 16 districts, thus increasing government‐led financing. No explicit reference to trauma or burn care financing

#### 3.1.2 Quality Appraisal Outcomes

The six peer‐reviewed studies included in this scoping review demonstrated varying levels of methodological rigor (Tables S6 to S9 [Supporting file]). Among the four quantitative non‐randomized studies, all met the MMAT criteria for having clear research questions, appropriate outcome measurements, consistent administration of exposure or intervention, and consideration of confounders (Gallaher et al., 2015; Purcell et al., 2020; Twea et al., 2024; Mody et al., 2023). However, outcome completeness was unclear in two studies (Gallaher et al., 2015; Purcell et al., 2020). Participant representativeness emerged as a notable limitation, with two studies rated as “Can't tell” for this criterion (Purcell et al., 2020; Twea et al., 2024), while one study did not meet the representativeness requirement (Mody et al., 2023).

The remaining two quantitative descriptive studies satisfied all MMAT criteria, demonstrating high methodological quality across sampling strategy, representativeness, appropriateness of measurements, and statistical analysis (Whitaker et al., 2024a; Whitaker et al., 2024b).

The 10 gray literature sources were appraised using the AACODS checklist (Table S10 [Supporting file]). All documents demonstrated strong authority, being produced by reputable international organizations, government institutions, or established non‐governmental actors, and authored by individuals or teams with relevant professional expertise. Most sources demonstrated clear aims and appropriate methodological or analytical approaches. However, the absence of detailed reference lists or bibliographies was a common limitation, observed in approximately three‐quarters of the documents.

In terms of objectivity and coverage, most gray literature sources presented balanced information with clearly articulated scope and author standpoint. Two documents showed limited evidence of balance (Interburns, 2019; Trent‐Gurbuz, 2018), reflecting their advocacy or program‐focused nature. While the majority of sources were current and relevant, a small number did not clearly specify dates directly related to content. Despite these limitations, all gray literature sources were judged to be highly significant, contributing essential contextual, policy, and financing insights that complemented the peer‐reviewed evidence.

Importantly, no studies or documents were excluded on the basis of quality appraisal. Instead, appraisal findings were used to support cautious interpretation of results, in line with best practice for scoping reviews.

#### 3.1.3 Research Techniques

Different techniques (or methods) were used by the studies to identify the real costs of trauma and burns treatment and prevention from both the health system and patient perspectives. Half of the studies utilized survey‐based methodology involving primary data collection [11, 39, 42]. Two did activity‐based costing [38, 40], one used mixed methodology involving hospital‐level costs and time‐driven activity‐based costing [41]. These methods show strong emphasis on population‐level knowledge and health systems research, revealing both service users and providers perspectives.

On the other hand, the gray literature utilized policy analysis and expert consultation [31, 35] literature review and economic forecasts/revenue projections along with consultation [28, 32], fiscal planning and budget analysis [28], economic and statistical analysis, and stakeholder and sectorial reports [30], descriptive reporting [37]. One utilized mixed‐methodology [34].

#### 3.1.4 Study Population

Diverse population groups were sampled in the studies reviewed. The majority (66.7%) were conducted in hospital settings, and they sampled trauma patients with varying characteristics, such as patients admitted for burns [38], patients with all types of trauma (dead or alive) [40], patients receiving orthopedic services [41], as well as operative and non‐operative adults with femoral shaft fractures [42].

One study sampled participants from the general population using the Health and Demographic Surveillance Site [11], and one was among health facility staff involved in injury care [39].

Only three of the gray literature included in the review specified the population targeted in the document [34, 36, 37]; they include trauma patients presenting to selected hospitals [34, 37], and trainee nurses not research subjects [36].

#### 3.1.5 Sample Characteristics

The reviewed studies were spread across pediatric, adult, and general populations, with many reporting male preponderances. The proportion of males in the samples ranged from 55% to 81%. The age distribution also varied, although some did not specify age data in their report [39, 40, 41]. Some reported a median age at injury of 22 years (IQR 10–40), indicating a broad age range comprising both children and adults [11]. Another study sampled mostly children, with 80% of patients under 18 years, the median age of 3 years was reported [38], compared to Mody et al.'s study, where adults with femoral fractures were targeted and a mean age of 36 years was recorded [42]. The included gray literature failed to report the characteristics of their sample.

#### 3.1.6 Sample Size

Varied sample sizes were reported across the six studies reviewed, ranging between 42 and 12,616 persons, while the mean sample size and median sample size were 2796 and 760, respectively. The largest sample were recorded in a study that reviewed hospital‐based data [40] while the smallest sample was reported by Mody et al. [42] in a socioeconomic impact survey, which was a sub‐study within a larger prospective cohort.

Just one of the gray literature indicated the sample size of over 118,013 trauma cases, which were derived from the registry dataset across 10 facilities in Malawi [34].

#### 3.1.7 Sources of Financing

Neither of the reviewed studies mentioned any private, donor, or external grant funding for trauma and burns prevention and control in Malawi. Perhaps because Malawi's healthcare system is fully subsidized by the government, all trauma and burns‐related financing is government‐funded, as some of the studies explicitly reported [11, 38]. Similarly, none of the gray literature reported financing mechanisms for trauma and burns prevention. Only two mentioned non‐financial support for burns prevention and care, including capacity building, medical supplies, and covering medical expenses (amount not specified) [36, 37].

#### 3.1.8 Type of Cases Investigated/Reported

The studies examined different types of trauma and burn cases over diverse time periods varying from 3 months to 3 years. Five out of six (83.3%) investigated traumatic cases [11, 39, 40], including fatal and non‐fatal injuries [11], orthopedic injuries [41], and femoral shaft fractures [42]. Only one (16.7%) assessed burn injuries [38]. On the other hand, three of the gray literature focused on burn [35, 36, 37], one documented multiple trauma types, such as RTC injuries, falls, and other trauma [34], while the rest specified neither trauma nor burns.

#### 3.1.9 Sources of Trauma and Burns Prevention and Care Financing

The current review indicated that there is a lack empirical finding on the financing of trauma and burns prevention in Malawi as none of the included studies analyzed financing using the revenue, pooling, and purchasing components. The included studies only identified the sources of trauma and burn care financing. It was noted that trauma and burns care/control in the country was dominated by the public sector, none reported funding from private or foreign donors. Some of the studies confirmed that healthcare service in Malawi is largely publicly funded through government allocations and non‐financial support from donors, with government and mission (Christian Health Association of Malawi) facilities being the primary providers. Care at these facilities is intended to be free at the point of use [11, 41].

Similarly, sources of finance for trauma and burns prevention and care were not indicated in the gray literature. Forms of support provided are mainly non‐financial, such as capacity building/training of health personnel, provision of donated medical supplies to facilities, and supporting indigent patients with an unspecified amount to cover medical bills [36, 37]. In addition, from both empirical and gray literature, there is no evidence of specific funding dedicated to trauma and burn prevention and care in Malawi. Rather, these are financed through the general health‐sector allocations.

#### 3.1.10 Direct Costs of Trauma and Burns Prevention and Care

The review found no empirical evidence on the direct costs of trauma and burn prevention. However, it was indicated that trauma and burn care in Malawi demand substantial financial and material resources, with costs dependent on the type of injury and hospital of care. A study assessing the diagnosis‐specific cost of orthopedic services found that at the tertiary level, personnel expenses accounted for the largest portion of costs (48%), followed by medicines and supplies (37%). For total expenditure, personnel costs represented the largest share at 30%, with medicines and supplies accounting for 23% [41]. Whereas for burn care, facility expenditures made up 30% of total costs, followed by medications (17.5%), human resources (16.6%), and clinical consumables (15.5%) [38].

The length of hospital stay emerged as the most significant factor influencing overall costs of orthopedic services [41]. At tertiary hospitals such as Mzuzu Central Hospital (MCH) and Kamuzu Central Hospital (KCH), total annual orthopedic departmental costs were US$545,254 and US$838,540, respectively. Inpatient care was found to be more expensive (US$43–US$53 per episode) than outpatient services (US$14–US$18). Direct orthopedic service delivery consumed a major fraction of aggregate costs (between 66% and 77%). The costs of treatment also varied by fracture type, with proximal ulna fractures being the most expensive at an average of US$714, while supracondylar fractures had the lowest mean cost of US$195 [41].

A cost analysis of burns care revealed that comprehensive inpatient burn care can be delivered in Malawi at a substantially lower cost than in other locations. The mean cost per inpatient admission was US$559.85 (SD ± US$736.17), with a mean daily per‐patient cost of US$24.26 (SD ± US$6.44) and a mean daily cost per 1% total burn surface area (TBSA) of US$2.65 (SD ± US$3.01). Facility expenditures accounted for the largest part of total costs (30%), followed by medications (17.5%), human resources (16.6%), and clinical consumables (15.5%). Analgesics and antibiotics constituted the majority of medication costs, while debridement and skin grafting procedures constituted most of the operative costs [38]. However, the included gray literature did not document the direct costs of trauma and burns prevention and care in Malawi.

#### 3.1.11 Indirect Cost of Trauma and Burns Prevention and Care

Despite Malawi's policy of free care in government facilities, indirect, out‐of‐pocket expenses were a major barrier to accessing timely and appropriate care for some segments of the population [11, 39, 42]. Trauma imposes significant socio‐economic burdens on patients and their households as families incur an average of US$2.87 on transportation per hospital visit [42]. In another study, approximately 5.6% of trauma cases failed to present for medical care after injury due to financial constraints, while 13.4% cited transportation costs due to long distance to medical facilities and poor state of road [11].

The reviewed studies indicate that even when direct medical costs are minimal, socio‐economic costs‐both direct (out‐of‐pocket expenses) and indirect (transportation, lost income, and opportunity costs, informal payments)‐were consistently recognized as major obstacles throughout the injury care pathway. These costs may impact healthcare access, especially for poor and rural residents [11, 39]. While indirect cost of trauma and burn care was documented, none of the studies revealed the same for the prevention of trauma and burn in Malawi. In addition, the gray literature made no mention of indirect costs of trauma and burns care and prevention.

#### 3.1.12 Total Startup Cost of Trauma Surveillance Registry

One of the studies revealed that establishing and maintaining a hospital‐based trauma registry in Malawi entails significant financial and human resource investment. The cost of setting up a trauma registry at Kamuzu Central Hospital was approximately US$3,196.24. This includes personnel, database initiation, staff training, and office supplies costs. Recurrent costs were estimated to amount to US$33,361.64, annually. In 2018, it cost US$2.64 per trauma patient registered [40].

Out of the total recurrent annual cost, personnel salaries constituted the biggest fraction (US$29,697.24 yearly), followed by costs of internet, facility space, supplies, and ongoing training. To ensure 24/7 data collection and data quality, trauma surveillance requires dedicated staff (including a manager, data clerk manager, and five full‐time data clerks), continuous training, and infrastructure [40].

#### 3.1.13 Challenges of Trauma and Burn Prevention and Care Financing

The reviewed studies highlighted some major challenges affecting financing of trauma and burn care in Malawi. These include insufficient public health funding, which limits dedicated trauma/burn‐specific investment, high service delivery costs, especially personnel and supply costs, reliance on unaccounted donor support, inequitable resource distribution, and a lack of cost‐effectiveness data to guide funding priorities. The lack of economic evaluation literature for different trauma‐related diagnoses also makes it difficult for policymakers to justify or prioritize financing for specific trauma or burn interventions [11, 38, 39, 41].

At the individual level, there was the problem of dependency on out‐of‐pocket and informal payments particularly in private facilities, long inpatient stays [39, 41], which increases the financial cost of care [11, 39], high perceived and actual cost of seeking care such as transport, food, and lost income, which discourages patients from accessing trauma/burn services [39, 42].

Notably, a major dearth in the reviewed gray literature is the lack of information on health financing for trauma and burn. Even the documents that reported healthcare financing within the main budget/fiscal planning did not mention specific budget allocations dedicated to trauma and burn care and prevention in Malawi.

## 4 Discussion

This scoping review provides the first comprehensive examination of trauma and burns financing in Malawi, revealing critical insights into the economic burden, financing mechanisms, and barriers to prevention and care in this low‐resource setting. The identification of only six studies meeting inclusion criteria underscores a substantial knowledge gap regarding trauma and burns financing in Malawi, which is concerning given that trauma represents a leading cause of morbidity and mortality in sub‐Saharan Africa [43, 44]. This paucity contrasts sharply with the extensive literature on trauma financing in high‐income countries, where comprehensive health economics evaluations are routine [45, 46]. The predominant focus on cost analysis (66.7% of studies) rather than comprehensive financing mechanisms in our review suggests that current research efforts in Malawi are reactive rather than proactive, differing from systematic approaches observed in countries like South Africa and Kenya where trauma financing research has been more comprehensive [47, 48], such as using the revenue, pooling, and purchasing components all of which are absent in the included studies we analyzed in this review.

A striking finding of our review is the complete reliance on general health‐sector allocations from the government, with no specific dedicated funding for trauma and burns care, in addition to lack of private sector involvement or external donor support specifically targeted at these services. This differs markedly from other health areas in Malawi, such as HIV/AIDS, tuberculosis, and maternal health, which benefit from substantial dedicated donor funding [49, 50]. This pattern also contrasts with trauma financing in other low‐ and middle‐income countries (LMICs) where public‐private partnerships and international funding play significant roles [51, 52]. While Malawi's policy of free healthcare at the point of service aligns with universal health coverage principles, the complete dependence on government funding raises concerns about financial sustainability similar to those documented in other sub‐Saharan African countries [53, 54].

The review reveals that trauma and burns care impose substantial direct costs on the healthcare system, with personnel costs constituting the largest proportion of expenditure (30–48% across studies). This finding is consistent with international literature showing that human resources represent the major cost component in trauma care globally [46]. However, the absolute costs identified in Malawi (US$195–US$714 for orthopedic procedures, US$559.85 for burn admissions) are substantially lower than those reported in high‐income countries, where trauma care costs can exceed US$10,000 per episode [45, 55]. This cost differential reflects lower labor costs and reduced resource intensity rather than more efficient care delivery, as suggested by the significant infrastructure constraints identified across studies.

Despite the policy of free care at government facilities, the review consistently identified significant financial barriers to trauma and burns care, with 5.6% of trauma cases failing to seek medical care due to financial constraints. This finding aligns with broader literature on healthcare access in sub‐Saharan Africa, where indirect costs often represent the primary barrier to care utilization even in systems with nominal free services [56, 57]. The transportation costs averaging US$2.87 per hospital visit identified in this review, while seemingly modest, represent a substantial proportion of daily income for many Malawians living below the poverty line, similar to patterns documented in other LMICs [17, 58]. The socioeconomic impact extends beyond immediate healthcare costs, with 29% of patients reporting decreased household income and 49% experiencing food insecurity following femoral shaft fractures, echoing findings from other trauma studies in low‐resource settings that demonstrate catastrophic health expenditures despite nominally free care [59, 60].

The substantial investment required for trauma surveillance systems (US$3,196.24 for setup and US$33,361.64 annually) demonstrates the hidden infrastructure costs essential for quality trauma care. These costs are comparable to those reported for trauma registry implementation in other LMICs, where startup costs typically range from US$2,000–US$5,000 and annual operating costs from US$25,000–US$50,000 [61]. However, the cost per patient registered (US$2.64) in Malawi appears favorable compared to similar systems in other African countries, suggesting relatively efficient data collection processes [62].

The emphasis on “Delay 3” (receiving care) as the most critical barrier according to healthcare staff differs from patterns observed in some other African settings, where transportation and financial barriers (Delays 1 and 2) are often prioritized [63, 64]. This finding suggests that while Malawi has made progress in improving access to healthcare facilities, significant capacity constraints remain at the facility level, similar challenges have been documented across the region [64].

The complete absence of donor funding for trauma and burns care identified in this review contrasts with the substantial international support for other health priorities in Malawi and represents a missed opportunity for system strengthening. This pattern reflects the priorities of global health funding, which has historically favored infectious diseases over injury prevention and trauma care [51, 65]. Recent initiatives such as the World Health Organization Emergency Care Systems Framework and the Lancet Commission on Global Surgery have launched initiatives to address this gap; however, translation into sustained funding mechanisms remains limited [52, 66].

## 5 Limitations

Several limitations of this review need to be acknowledged. The small number of included studies limits generalizability and may not capture the full scope of trauma and burns financing challenges in Malawi. The predominant focus on hospital‐based studies may underestimate the true burden, as many cases may not reach formal healthcare facilities due to financial or geographical barriers, a phenomenon well‐documented in other LMICs [17]. The lack of comprehensive costing studies that include both direct and indirect costs from societal perspectives represents a significant knowledge gap compared to more mature health economics literature in high‐income countries [45, 46].

## 6 Conclusion

Future research priorities should include comprehensive cost‐effectiveness evaluations of prevention and treatment interventions, assessment of alternative financing mechanisms, including insurance schemes and public–private partnerships, and broader costing studies that capture the full societal impact of trauma and burns. Additionally, comparative studies with other countries in the region could provide valuable insights for policy development and resource allocation optimization.

## Author Contributions

**Kafayat Aminu:** conceptualization, data curation, formal analysis, funding acquisition, investigation, methodology, validation, visualization, project administration, resources, software, supervision, writing – original draft, writing – review and editing. **Oluwatobi Emmanuel Adegbile:** data curation, resources, validation, writing – original draft, writing – review and editing. **Olubukola Omobowale:** resources, writing – original draft. **Yovanthi Anurangi Jayasinghe:** investigation, methodology, validation, visualization, resources, writing – original draft, writing – review and editing. **Adetayo Olorunlana:** resources, writing – original draft. **Precious Chika Nnannah:** conceptualization, data curation, investigation, methodology, project administration, resources, software, validation, visualization, writing – original draft. **Emeka Okeke:** resources, validation, visualization, writing – original draft. **Ugochukwu Anthony Eze:** resources, writing – review and editing. **Afeez Abolarinwa Salami:** resources, investigation, methodology. **Rita Amarachi Nwebo:** conceptualization, data curation, investigation, methodology, project administration, resources, software, validation, visualization, writing – original draft. **Kehinde Kazeem Kanmodi:** conceptualization, data curation, funding acquisition, investigation, methodology, visualization, project administration, resources, software, supervision, validation, writing – original draft, writing – review and editing. [Correction added on 19 May 2026, after first online publication: Author Contributions section has been modified.]

## Ethics Statement

This study did not collect data from human or animal subjects but an open research repository.

## Conflicts of Interest

Kehinde Kazeem Kanmodi is an Editorial Board member of *Health Science Reports* and co‐authors of this article. To minimize bias, he was excluded from all editorial decision‐making related to the acceptance of this article for publication. Other authors have no conflict of interest to declare.

## Transparency Statement

The lead author, Kehinde Kazeem Kanmodi, affirms that this manuscript is an honest, accurate, and transparent account of the study being reported; that no important aspects of the study have been omitted; and that any discrepancies from the study as planned (and, if relevant, registered) have been explained.

## Supporting information

Supporting File

## Data Availability

The data that support the findings of this study are available in the supporting material of this article.

## References

[1] S. Kasenda , D. Mategula , G. E. Manda , and T. K. Chokotho , “Risk Factors for Mortality Among Hospitalised Adult Burn Patients at a Malawian Tertiary Hospital Burns Unit,” East and Central African Journal of Surgery 24, no. 2 (2019): 124–132.

[2] S. Kasenda , D. Mategula , and T. Chokotho , “Burns Among Adults in a Major Malawian Burn Unit: Epidemiology and Factors Associated With Prolonged Hospital Stay,” Malawi Medical Journal 35, no. 3 (2023): 132–140. 38362289 10.4314/mmj.v35i3.1 PMC10865059

[3] H. S. Richards , R. M. T. Staruch , S. Kinsella , et al., “Top Ten Research Priorities in Global Burns Care: Findings From the James Lind Alliance Global Burns Research Priority Setting Partnership,” Lancet Global Health 13, no. 6 (2025): e1140–e1150. 40286806 10.1016/S2214-109X(25)00059-2

[4] S. Peiffer , L. Kayange , S. An , O. Boddie , A. Charles , and J. Gallaher , “The Treatment Effect of Operative Intervention for Flame Versus Scald Burns in Resource‐Limited Settings,” Burns 50, no. 9 (2024): 107248. 39447288 10.1016/j.burns.2024.08.014 PMC11625598

[5] E. L. Peter , S. F. Rumisha , K. O. Mashoto , O. M. Minzi , and S. Mfinanga , “Efficacy of Standardized Extract of Hibiscus Sabdariffa L. (Malvaceae) in Improving Iron Status of Adults in Malaria Endemic Area: A Randomized Controlled Trial,” Journal of Ethnopharmacology 209 (2017): 288–293. 28755971 10.1016/j.jep.2017.07.037

[6] J. Samuel , E. Campbell , S. Mjuweni , A. Muyco , B. Cairns , and A. Charles , “The Epidemiology, Management, Outcomes and Areas for Improvement of Burn Care in Central Malawi: An Observational Study,” Journal of International Medical Research 39, no. 3 (2011): 873–879. 21819720 10.1177/147323001103900321 PMC3290411

[7] E. Broadis , T. Chokotho , D. Mackay , and E. Germeni , “First Aid Management of Paediatric Burn and Scald Injuries in Southern Malawi: A Mixed Methods Study,” Burns 46, no. 3 (2020): 727–736. 31732221 10.1016/j.burns.2019.08.015

[8] J. C. Samuel , E. L. P. Campbell , A. G. Charles , and B. A. Cairns , “Burn Epidemiology and Burn Care in Malawi: Outlining Prevention Strategies,” Injury Prevention 16, no. Suppl 1 (2010): A61.

[9] World Bank , Trauma Incidence and Emergency Medical Services in Malawi [Internet] (The World Bank, 2022), https://thedocs.worldbank.org/en/doc/425609b15161f83b64a832dbd0bcba1a-0050022021/original/15996-Policy-Brief-TRA-Malawi-Trauma-WEB.pdf.

[10] K. J. A. Harding , K. Mody , L. M. Amlani , et al., “Technical Priorities for Orthopaedic Trauma Care Development in Malawi,” Malawi Medical Journal 36, no. 3 (2024): 185–207. 40018394 10.4314/mmj.v36i3.5 PMC11862851

[11] J. Whitaker , A. S. Amoah , A. Dube , R. Rickard , A. J. M. Leather , and J. Davies , “Access to Quality Care After Injury in Northern Malawi: Results of a Household Survey,” BMC Health Services Research 24, no. 1 (2024): 131. 38268016 10.1186/s12913-023-10521-8 PMC10809521

[12] F. Botelho , E. Reis , K. Ribeiro , et al., “Decolonizing Global Surgery: Bethune Round Table, 2022 Conference on Global Surgery (Virtual), June 16–18, 2022,” Canadian Journal of Surgery 65, no. 4 Suppl 1 (2022): S1–S18. 10.1503/cjs.007622 PMC9388215 35961679

[13] L. B. Ngoie , Musculoskeletal Impairment and Road Traffic Injuries in Malawi. 2023 [cited 2025 Jun 16], https://bora.uib.no/bora-xmlui/handle/11250/3082446.

[14] O. K. Oyedele and T. V. Lawal , “Global Dominance of Non‐Institutional Delivery and the Risky Impact on Maternal Mortality Spike in 25 Sub‐Saharan African Countries,” Global Health Research and Policy 10, no. 1 (2025): 10. 40012017 10.1186/s41256-025-00409-x PMC11866781

[15] L. N. Purcell , J. Sincavage , W. Banda , et al., “The Effect of Burn Mechanism on Pediatric Mortality in Malawi: A Propensity Weighted Analysis,” Burns 47, no. 1 (2021): 222–227. 33277092 10.1016/j.burns.2019.12.018 PMC7855906

[16] J. V. E. Gerstl , A. N. Ehsan , P. Lassarén , et al., “The Global Macroeconomic Burden of Burn Injuries,” Plastic & Reconstructive Surgery 153, no. 3 (2024): 743–752. 37093034 10.1097/PRS.0000000000010595

[17] M. Kotagal , K. J. Agarwal‐Harding , C. Mock , R. Quansah , C. Arreola‐Risa , and J. G. Meara , “Health and Economic Benefits of Improved Injury Prevention and Trauma Care Worldwide,” PLoS One 9, no. 3 (2014): e91862. 24626472 10.1371/journal.pone.0091862 PMC3953529

[18] D. Geehan , “The Big Hurt: Trauma System Funding in Today's Health Care Environment,” Missouri Medicine 107, no. 5 (2010): 324–327. PMID 21207783; PMCID: PMC6188402. 21207783 PMC6188402

[19] World Health Organization Health Financing Fact Sheet, [accessed on March 2, 2026; 16:01pm WAT, www.who.int.

[20] S. L. Hsu , editor. Valuing Health For Environmental Policy With Special Emphasis on Children's Health Issues. 1999 [cited 2025 Jun 16], https://19january2021snapshot.epa.gov/sites/static/files/2017-09/documents/ee-0416-01.pdf.

[21] M. Cropper , E. Joiner , and A. Krupnick , “Revisiting EPA's Value Per Statistical Life,” Review of Environmental Economics and Policy 18, no. 2 (2024): 193–211.

[22] W. K. Viscusi , “Extending the Domain of the Value of a Statistical Life,” Journal of Benefit‐Cost Analysis 12, no. 1 (2021): 1–23.

[23] K. Croke , L. Chokotho , S. Milusheva , et al., “Implementation of a Multi‐Center Digital Trauma Registry: Experience in District and Central Hospitals in Malawi,” International Journal of Health Planning and Management 35, no. 5 (2020): 1157–1172. 32715521 10.1002/hpm.3023

[24] H. Arksey and L. O'Malley , “Scoping Studies: Towards a Methodological Framework,” International Journal of Social Research Methodology 8 no.1 (2005): 19–32, https://www.tandfonline.com/doi/abs/10.1080/1364557032000119616.

[25] M. J. Page , J. E. McKenzie , P. M. Bossuyt , et al., “The PRISMA 2020 Statement: An Updated Guideline for Reporting Systematic Reviews,” BMJ 372 (2021): n71. 33782057 10.1136/bmj.n71 PMC8005924

[26] Q. N. Hong , P. Pluye , S. Fàbregues , et al. Mixed Methods Appraisal Tool (MMAT), Version 2018: User Guide [Internet]. Canada: Canadian Intellectual Property Office, Industry Canada; 2018 [cited 2025 Jun 16]. 1–11, https://bmjopen.bmj.com/content/bmjopen/11/2/e039246/DC3/embed/inline-supplementary-material-3.pdf.

[27] M. A. O'Donovan , P. McCallion , M. McCarron , L. Lynch , H. Mannan , and E. Byrne , “A Narrative Synthesis Scoping Review of Life Course Domains Within Health Service Utilisation Frameworks,” HRB Open Research 2 (2019): 6, https://pubmed.ncbi.nlm.nih.gov/32296746/. 32296746 10.12688/hrbopenres.12900.1 PMC7140772

[28] Republic of Malawi, Ministry of Finance and Economic Affairs , The 2025/2026 Budget Policy statement [Internet]. Republic of Malawi: Ministry of Finance and Economic Affairs; 2025 [cited 2026 Oct 2], https://www.finance.gov.mw/documents/uploads/2026-01/Budget%20Policy%20Statement%202025-26_0.pdf.

[29] Republic of Malawi, Ministry of Finance and Economic Affairs , Draft 2025/26 financial statement. [Internet]. Republic of Malawi: Ministry of Finance and Economic Affairs; 2025, https://www.finance.gov.mw/documents/uploads/2026-01/Financial%20Statement%202025-26.pdf.

[30] Republic of Malawi, Ministry of Finance and Economic Affairs , Annual Economic Report 2025 [Internet]. Republic of Malawi: Ministry of Finance and Economic Affairs; 2026, https://finance.gov.mw/documents/uploads/2026-01/Annual%20Economic%20Report%202025.pdf.

[31] International Monetary Fund , Malawi: Economic Development Document [Internet]. Washington, D.C.; 2017 [cited 2026 Feb 9] p. 1. Report No.: 17/184, https://elibrary.imf.org/openurl?genre=journal&issn=1934-7685&volume=2017&issue=184.

[32] Republic of Malawi, Ministry of Finance and Economic Affairs , Regional Climate Resilience Program for Eastern and Southern Africa: Series of Projects 2 (P181308) [Internet]. Malawi: Ministry of Finance and Economic Affairs; 2023 [cited 2026 Feb 10] p. 298. Report No.: P181308, https://documents1.worldbank.org/curated/en/099110823152512414/pdf/P1813080dc4197024091eb0c3960c185fba.pdf.

[33] World Health Organization , WHO Malawi Country Office Comprehensive Annual Report 2024 [Internet]. WHO Regional Office for Africa: World Health Organization; 2025 [cited 2026 Oct 2], https://www.afro.who.int/sites/default/files/2025-10/WHO%20MALAWI%202024%20ANNUAL%20REPORT_0.pdf.

[34] The World Bank , Trauma Incidence and Emergency Medical Services in Malawi [Internet]. Washington DC: The World Bank; 2021 [cited 2026 Oct 2], https://documents1.worldbank.org/curated/en/176181642602064254/pdf/Trauma-Incidence-and-Emergency-Medical-Services-in-Malawi.pdf.

[35] World Health Organization , A WHO plan for burn prevention and care [Internet]. Mock C, Peck M, Peden M, Krug E, editors. Geneva, Switzerland: World Health Organization; 2008 [cited 2026 Feb 10], https://www.who.int/publications/i/item/9789241596299.

[36] Interburns. Interburns , 2019 [cited 2026 Feb 9]. Wales for Africa Fund Training for Burn Nurses, https://interburns.org/news/wales-for-africa.

[37] C. J. Trent‐Gurbuz , School of Medicine and Health Sciences. USA: Africa Burn Relief Program; 2018 [cited 2026 Feb 10]. Africa Burn Relief Program, https://smhs.gwu.edu/news/africa-burn-relief-program.

[38] J. R. Gallaher , S. Mjuweni , B. A. Cairns , and A. G. Charles , “Burn Care Delivery in a Sub‐Saharan African Unit: A Cost Analysis Study,” International Journal of Surgery 19 (2015): 116–120. 26003120 10.1016/j.ijsu.2015.05.015

[39] J. Whitaker , T. Njawala , V. Nyirenda , et al., “Identifying and Prioritising Barriers to Injury Care in Northern Malawi, Results of a Multifacility Multidisciplinary Health Facility Staff Survey,” PLoS One 19 9 (2024): e0308525. 39264901 10.1371/journal.pone.0308525 PMC11392338

[40] L. N. Purcell , E. Nip , J. Gallaher , C. Varela , Y. Gondwe , and A. Charles , “Design and Implementation of a Hospital‐Based Trauma Surveillance Registry in a Resource‐Poor Setting: A Cost Analysis Study,” Injury() 51, no. 7 (2020): 1548–1553. 32456956 10.1016/j.injury.2020.04.044 PMC7372905

[41] P. Twea , D. Watkins , O. F. Norheim , et al., “The Economic Costs of Orthopaedic Services: A Health System Cost Analysis of Tertiary Hospitals in a Low‐Income Country,” Health Economics Review 14, no. 1 (2024): 13. 38367132 10.1186/s13561-024-00485-8 PMC10874068

[42] K. S. Mody , H. H. Wu , L. C. Chokotho , et al., “The Socioeconomic Consequences of Femoral Shaft Fracture for Patients in Malawi,” Malawi Medical Journal 35, no. 3 (2023): 141–150. 38362293 10.4314/mmj.v35i3.2 PMC10865065

[43] D. Adeloye , K. Bowman , K. Y. Chan , S. Patel , H. Campbell , and I. Rudan , “Global and Regional Child Deaths Due to Injuries: An Assessment of the Evidence,” Journal of Global Health 8, no. 2 (2018): 021104, 10.7189/jogh.08.021104. 30675338 PMC6317703

[44] J. A. Haagsma , N. Graetz , I. Bolliger , et al., “The Global Burden of Injury: Incidence, Mortality, Disability‐Adjusted Life Years and Time Trends From the Global Burden of Disease Study 2013,” Injury Prevention 22, no. 1 (2016): 3–18. 26635210 10.1136/injuryprev-2015-041616 PMC4752630

[45] B. J. Gabbe , R. A. Lyons , J. E. Harrison , et al., “Validating and Improving Injury Burden Estimates Study: The Injury‐Vibes Study Protocol,” Injury Prevention 20, no. 3 (2014): e4. 23920023 10.1136/injuryprev-2013-040936

[46] S. Polinder , J. A. Haagsma , H. Toet , and E. F. van Beeck , “Epidemiological Burden of Minor, Major and Fatal Trauma in a National Injury Pyramid,” British Journal of Surgery 99, no. Suppl_1 (2011): 114–120. 10.1002/bjs.7708 22441864

[47] W. Cholo , W. Odero , and J. Ogendi , “The Burden of Motorcycle Crash Injuries on the Public Health System in Kisumu City, Kenya,” Global Health: Science and Practice 11, no. 1 (2023): e2200197. 36853633 10.9745/GHSP-D-22-00197 PMC9972383

[48] T. C. Hardcastle and P. Brysiewicz , “Trauma Care in South Africa: From Humble Beginnings to An Afrocentric Outreach,” International Emergency Nursing 21, no. 2 (2013): 118–122. 23615519 10.1016/j.ienj.2012.05.002

[49] C. Chansa , J. Sundewall , D. McIntyre , G. Tomson , and B. C. Forsberg , “Exploring SWAp's Contribution to the Efficient Allocation and Use of Resources in the Health Sector in Zambia,” Health Policy and Planning 23, no. 4 (2008): 244–251. 18562459 10.1093/heapol/czn013

[50] M. T. Makwero , “Delivery of Primary Health Care in Malawi,” African Journal of Primary Health Care & Family Medicine 10, no. 1 (2018): e1–e3, 10.4102/phcfm.v10i1.1799. PMC6018651 29943590

[51] C. Mock , M. Joshipura , C. Arreola‐Risa , and R. Quansah , “An Estimate of the Number of Lives That Could Be Saved Through Improvements in Trauma Care Globally,” World Journal of Surgery 36, no. 5 (2012): 959–963. 22419411 10.1007/s00268-012-1459-6

[52] T. A. Reynolds , B. Stewart , I. Drewett , et al., “The Impact of Trauma Care Systems in Low‐ and Middle‐Income Countries,” Annual Review of Public Health 38, no. 1 (2017): 507–532. 10.1146/annurev-publhealth-032315-021412 28125389

[53] G. Lagomarsino , A. Garabrant , A. Adyas , R. Muga , and N. Otoo , “Moving Towards Universal Health Coverage: Health Insurance Reforms in Nine Developing Countries in Africa and Asia,” Lancet 380, no. 9845 (2012): 933–943. 22959390 10.1016/S0140-6736(12)61147-7

[54] A. Mills , M. Ally , J. Goudge , J. Gyapong , and G. Mtei , “Progress Towards Universal Coverage: The Health Systems of Ghana, South Africa and Tanzania,” Health Policy and Planning 27, no. suppl 1 (2012): i4–i12. 22388499 10.1093/heapol/czs002

[55] E. J. MacKenzie , F. P. Rivara , G. J. Jurkovich , et al., “A National Evaluation of the Effect of Trauma‐Center Care on Mortality,” New England Journal of Medicine 354, no. 4 (2006): 366–378. 16436768 10.1056/NEJMsa052049

[56] M. E. Kruk , G. Mbaruku , C. W. McCord , M. Moran , P. C. Rockers , and S. Galea , “Bypassing Primary Care Facilities for Childbirth: A Population‐Based Study in Rural Tanzania,” Health Policy and Planning 24, no. 4 (2009): 279–288. 19304785 10.1093/heapol/czp011

[57] D. McIntyre , M. Thiede , G. Dahlgren , and M. Whitehead , “What Are the Economic Consequences for Households of Illness and of Paying for Health Care in Low‐ and Middle‐Income Country Contexts?,” Social Science & Medicine 62, no. 4 (2006): 858–865. 16099574 10.1016/j.socscimed.2005.07.001

[58] C. Arsenault , P. Fournier , A. Philibert , et al., “Emergency Obstetric Care in Mali: Catastrophic Spending and Its Impoverishing Effects on Households,” Bulletin of the World Health Organization 91, no. 3 (2013): 207–216. 23476093 10.2471/BLT.12.108969 PMC3590618

[59] D. O. Abegunde , C. D. Mathers , T. Adam , M. Ortegon , and K. Strong , “The Burden and Costs of Chronic Diseases in Low‐Income and Middle‐Income Countries,” Lancet 370, no. 9603 (2007): 1929–1938. 18063029 10.1016/S0140-6736(07)61696-1

[60] H. Nguyen , R. Ivers , S. Jan , A. Martiniuk , and C. Pham , “Catastrophic Household Costs Due to Injury in Vietnam,” Injury() 44, no. 5 (2013): 684–690. 22658420 10.1016/j.injury.2012.05.006

[61] M. Labinjo , C. Juillard , O. C. Kobusingye , and A. A. Hyder , “The Burden of Road Traffic Injuries in Nigeria: Results of a Population‐Based Survey,” Injury Prevention 15, no. 3 (2009): 157–162. 19494094 10.1136/ip.2008.020255

[62] M. Mutto , S. Lawoko , C. Nansamba , E. Ovuga , and L. Svanstrom , “Unintentional Childhood Injury Patterns, Odds, and Outcomes in Kampala City: An Analysis of Surveillance Data From the National Pediatric Emergency Unit,” Journal of Injury and Violence Research 3, no. 1 (2011): 13–18. 21483209 10.5249/jivr.v3i1.56 PMC3134920

[63] E. Calvello , T. Reynolds , J. M. Hirshon , et al., “Emergency Care in Sub‐Saharan Africa: Results of a Consensus Conference,” African Journal of Emergency Medicine 3, no. 1 (2013): 42–48.

[64] P. O. Ouma , J. Maina , P. N. Thuranira , et al., “Access to Emergency Hospital Care Provided by the Public Sector in Sub‐Saharan Africa in 2015: A Geocoded Inventory and Spatial Analysis,” Lancet Global Health 6, no. 3 (2018): e342–e350. 29396220 10.1016/S2214-109X(17)30488-6 PMC5809715

[65] R. Gosselin , “Injuries: The Neglected Burden in Developing Countries,” Bulletin of the World Health Organization 87, no. 4 (2009): 246. 19551225 10.2471/BLT.08.052290 PMC2672580

[66] J. G. Meara , A. J. M. Leather , L. Hagander , et al., “Global Surgery 2030: Evidence and Solutions for Achieving Health, Welfare, and Economic Development,” Lancet 386, no. 9993 (2015): 569–624. 25924834 10.1016/S0140-6736(15)60160-X

