# Correction to “Financing of Trauma and Burn Prevention and Care in Malawi: A Scoping Review”

**DOI:** 10.1002/hsr2.72547

**Published:** 2026-05-19

K. Aminu, O. E. Adegbile, O. Omobowale, et al., “Financing of Trauma and Burn Prevention and Care in Malawi: A Scoping Review,” Health Science Reports9 (2026): e72462, https://doi.org/10.1002/hsr2.72462.

In the Author Contributions section, the information was incomplete and should be updated as follows:

**Author Contributions**

**Kafayat Aminu:** conceptualization, data curation, formal analysis, funding acquisition, investigation, methodology, validation, visualization, project administration, resources, software, supervision, writing – original draft, writing – review and editing**. Oluwatobi Emmanuel Adegbile:** data curation, resources, validation, writing – original draft, writing – review and editing**. Olubukola Omobowale:** resources, writing – original draft. **Yovanthi Anurangi Jayasinghe:** investigation, methodology, validation, visualization, resources, writing – original draft, writing – review and editing. **Adetayo Olorunlana:** resources, writing – original draft. **Precious Chika Nnannah:** conceptualization, data curation, investigation, methodology, project administration, resources, software, validation, visualization, writing – original draft. **Emeka Okeke:** resources, validation, visualization, writing – original draft. **Ugochukwu Anthony Eze:** resources, writing – review and editing. **Afeez Abolarinwa Salami:** resources, investigation, methodology. **Rita Amarachi Nwebo:** conceptualization, data curation, investigation, methodology, project administration, resources, software, validation, visualization, writing – original draft. **Kehinde Kazeem Kanmodi:** conceptualization, data curation, funding acquisition, investigation, methodology, visualization, project administration, resources, software, supervision, validation, writing – original draft, writing – review and editing.

The online version of this article has been corrected accordingly.

We apologize for these errors.

