# Supplementary material for: Financing of Trauma and Burn Prevention and Care in Malawi: A Scoping Review
Source: Health Sci Rep. 2026 Apr 28;9(5):e72462. doi: 10.1002/hsr2.72462 (PMC13122117; doi:10.1002/hsr2.72462)
Supplement: Supplementary file 1 — Supporting File [file HSR2-9-e72462-s001.docx]

**SUPPLEMENTARY FILE**

**Table S1. Search string for PubMed database search**

| **Tag** | **Subject search** | **Search String** |
| --- | --- | --- |
| #1 | Financing | ((((((((((((money[Title/Abstract]) OR (grant*[Title/Abstract])) OR (fund*[Title/Abstract])) OR (sponsor*[Title/Abstract])) OR (financ*[Title/Abstract])) OR (loan[Title/Abstract])) OR (bank*[Title/Abstract])) OR (budget*[Title/Abstract])) OR (aid[Title/Abstract])) OR (donor[Title/Abstract])) OR (donat*[Title/Abstract])) OR (invest*[Title/Abstract])) OR (allocat*[Title/Abstract]) |
| #2 | Trauma and Burn | ((((((trauma*[Title/Abstract]) OR (accident*[Title/Abstract])) OR (injur*[Title/Abstract])) OR (fracture*[Title/Abstract])) OR (wound[Title/Abstract])) OR (burn[Title/Abstract])) OR (scald[Title/Abstract]) |
| #3 | Malawi | Malawi[Title/Abstract] |
| #4 | #1 AND #2 AND #3 | ((#1) AND (#2)) AND (#3) |

**Table S2. Search string for SCOPUS database search**

| **Tag** | **Subject search** | **Search String** |
| --- | --- | --- |
| #1 | Financing | ( TITLE-ABS-KEY ( money ) OR TITLE-ABS-KEY ( grant* ) OR TITLE-ABS-KEY ( fund* ) OR TITLE-ABS-KEY ( sponsor* ) OR TITLE-ABS-KEY ( financ* ) OR TITLE-ABS-KEY ( loan ) OR TITLE-ABS-KEY ( bank* ) OR TITLE-ABS-KEY ( budget* ) OR TITLE-ABS-KEY ( aid ) OR TITLE-ABS-KEY ( donor ) OR TITLE-ABS-KEY ( donat* ) OR TITLE-ABS-KEY ( invest* ) OR TITLE-ABS-KEY ( allocat* ) ) |
| #2 | Trauma and Burn | ( TITLE-ABS-KEY ( trauma* ) OR TITLE-ABS-KEY ( accident* ) OR TITLE-ABS-KEY ( injur* ) OR TITLE-ABS-KEY ( fracture ) OR TITLE-ABS-KEY ( wound ) OR TITLE-ABS-KEY ( burn ) OR TITLE-ABS-KEY ( scald ) ) |
| #3 | Malawi | TITLE-ABS-KEY ( malawi ) |
| #4 | #1 AND #2 AND #3 | ( TITLE-ABS-KEY ( malawi ) ) AND ( ( TITLE-ABS-KEY ( trauma* ) OR TITLE-ABS-KEY ( accident* ) OR TITLE-ABS-KEY ( injur* ) OR TITLE-ABS-KEY ( fracture ) OR TITLE-ABS-KEY ( wound ) OR TITLE-ABS-KEY ( burn ) OR TITLE-ABS-KEY ( scald ) ) ) AND ( ( TITLE-ABS-KEY ( money ) OR TITLE-ABS-KEY ( grant* ) OR TITLE-ABS-KEY ( fund* ) OR TITLE-ABS-KEY ( sponsor* ) OR TITLE-ABS-KEY ( financ* ) OR TITLE-ABS-KEY ( loan ) OR TITLE-ABS-KEY ( bank* ) OR TITLE-ABS-KEY ( budget* ) OR TITLE-ABS-KEY ( aid ) OR TITLE-ABS-KEY ( donor ) OR TITLE-ABS-KEY ( donat* ) OR TITLE-ABS-KEY ( invest* ) OR TITLE-ABS-KEY ( allocat* ) ) ) |

**Table S3. Search string for other database (AMED – The Allied and Complementary Medicine Database, CINAHL Ultimate, Dentistry and Oral Sciences Source, SPORTDiscus with Full Text, APA PsycArticles, Psychology and Behavioral Sciences Collection, Regional Business News, and APA PsycInfo) search via EBSCO interface**

| **Tag** | **Subject search** | **Search String** |
| --- | --- | --- |
| S1 | Financing | AB money OR AB grant* OR AB fund* OR AB sponsor* OR AB financ* OR AB loan OR AB bank* OR AB budget* OR AB aid OR AB donor OR AB donat* OR AB invest* OR AB allocat* |
| S2 | Trauma and Burn | AB Trauma* OR AB accident* OR AB injur* OR AB fracture* OR AB wound OR AB burn OR AB scald |
| S3 | Malawi | AB Malawi |
| S4 | S1 AND S2 AND S3 | S1 AND S2 AND S3 |

**Table S4. Search string for Google Scholar and websites of the World Health Organization and Malawi Ministry of Health**

| **Tag** | **Subject search** | **Search String (free text)** |
| --- | --- | --- |
| S1 | Financing | ("money" OR "grant" OR "fund" OR "sponsor" OR "finance" OR "loan" OR "bank" OR "budget" OR "aid" OR "donor" OR "donation" OR "invest" OR "allocation") |
| S2 | Trauma and Burn | ("trauma" OR "accident" OR "injury" OR "fracture" OR "wound" OR "burn" OR "scald") |
| S3 | Malawi | Malawi |
| S4 | S1 AND S2 AND S3 | ("money" OR "grant" OR "fund" OR "sponsor" OR "finance" OR "loan" OR "bank" OR "budget" OR "aid" OR "donor" OR "donation" OR "invest" OR "allocation") AND ("trauma" OR "accident" OR "injury" OR "fracture" OR "wound" OR "burn" OR "scald") AND Malawi |

**Table S5a. List of peer-reviewed literature considered for full text screening for** **Financing of Trauma and Burn Prevention and Control in Malawi**

| No. | Citation | Included | Excluded  (with reason) |
| --- | --- | --- | --- |
| 1 | Whitaker, J., Amoah, A.S., Dube, A. et al. Access to quality care after injury in Northern Malawi: results of a household survey. BMC Health Serv Res 24, 131 (2024a). https://doi.org/10.1186/s12913-023-10521-8 | Yes |  |
| 2 | Sundet, M., Mulima, G., Kajombo, C., Gjerde, H., Christophersen, A.S. and Young, S. (2020). Adult pedestrian and cyclist injuries in Lilongwe, Malawi: a cross-sectional study. Malawi medical journal, 32(4), pp.197-204. |  | Yes  (Wrong study outcome, and wrong study design) |
| 3 | Mody, K.S., Wu, H.H., Chokotho, L.C., Mkandawire, N.C., Young, S., Lau, B.C., Shearer, D. and Agarwal-Harding, K.J. (2023). The Socioeconomic consequences of femoral shaft fracture for patients in Malawi. Malawi Medical Journal, 35(3), pp.141-150. | Yes |  |
| 4 | Gallaher, J.R., Mjuweni, S., Cairns, B.A. and Charles, A.G. (2015). Burn care delivery in a sub-Saharan African unit: a cost analysis study. International Journal of Surgery, 19, pp.116-120. | Yes |  |
| 5 | Purcell, L.N., Nip, E., Gallaher, J., Varela, C., Gondwe, Y. and Charles, A. (2020). Design and implementation of a hospital-based trauma surveillance registry in a resource-poor setting: a cost analysis study. Injury, 51(7), pp.1548-1553. | Yes |  |
| 6 | Stevenson, J.H., Borgstein, E., Broadis, E., Chokotho, T. and Watson, S. (2021). Global surgery and burn injuries; experience from a 25 year partnership in Malawi. The Surgeon, 19(6), pp.338-343. |  | Yes (Wrong study objectives) |
| 7 | Ngwira, G.M., Bolaane, B. and Parida, B.P. (2023). Investigating the trend of road traffic fatalities in Malawi using Mann-Kendall statistic. Heliyon, 9(2). 10.1111/j.1600-0722.2010.00776.x. Epub 2010 Sep 30. PMID: 21083625. |  | Yes  (Wrong study outcome, and wrong study design) |
| 8 | Schade, A.T., Mbowuwa, F., Chidothi, P., MacPherson, P., Graham, S.M., Martin Jr, C., Harrison, W.J. and Chokotho, L. (2021). Epidemiology of fractures and their treatment in Malawi: Results of a multicentre prospective registry study to guide orthopaedic care planning. PLoS One, 16(8), p.e0255052. |  | Yes  (Wrong study outcome) |
| 9 | Whitaker, J., Njawala, T., Nyirenda, V., Amoah, A.S., Dube, A., Chirwa, L., Munthali, B., Rickard, R., Leather, A.J. and Davies, J. (2024b). Identifying and prioritising barriers to injury care in Northern Malawi, results of a multifacility multidisciplinary health facility staff survey. Plos one, 19(9), p.e0308525. | Yes |  |
| 10 | Schade, A.T., Nyamulani, N., Banza, L.N., Metcalfe, A.J., Leather, A., Madan, J.J., Lallloo, D.G., Harrison, W.J. and MacPherson, P. (2021). Protocol for a prospective cohort study of open tibia fractures in Malawi with a nested implementation of open fracture guidelines. Wellcome open research, 6, p.228. |  | Yes  (Wrong publication type, and Wrong study outcome) |
| 11 | Twea, P., Watkins, D., Norheim, O.F., Munthali, B., Young, S., Chiwaula, L., Manthalu, G., Nkhoma, D. and Hangoma, P. (2024). The economic costs of orthopaedic services: a health system cost analysis of tertiary hospitals in a low-income country. Health economics review, 14(1), p.13. | Yes |  |
| 12 | Gallaher, J.R., Banda, W., Robinson, B., Purcell, L.N. and Charles, A. (2020). Access to operative intervention reduces mortality in adult burn patients in a resource-limited setting in Sub-Saharan Africa. World journal of surgery, 44, pp.3629-3635. |  | Yes (Wrong study objectives) |
| 13 | Gallaher, J., Purcell, L.N., Banda, W., Reid, T. and Charles, A. (2021). The Association Between Burn Unit Census and Operative Intervention in a Resource-Limited Setting. World Journal of Surgery, 45, pp.1686-1691. |  | Yes (Wrong study objectives) |
| 14 | Davis, D., An, S., Kayange, L., Wolf, L., Boddie, O., Charles, A. and Gallaher, J. (2023). The timing of operative intervention for pediatric burn patients in Malawi. World Journal of Surgery, 47(12), pp.3093-3098. |  | Yes  (Wrong study outcome, and Wrong study design) |

**Table S5b. Grey literature considered for full-text screening for financing of trauma and burn prevention and care in Malawi**

| No. | Citation | Included | Excluded (with reason) |
| --- | --- | --- | --- |
| 1 | Interburns. Wales for Africa fund training for burn nurses. Interburns; 2019. https://interburns.org/news/wales-for-africa | Yes |  |
| 2 | Trent-Gurbuz CJ. Africa burn relief program. School of Medicine and Health Sciences, George Washington University; 2018. https://smhs.gwu.edu/news/africa-burn-relief-program | Yes |  |
| 3 | Mock C, Peck M, Peden M, Krug E, editors. A WHO plan for burn prevention and care. World Health Organization; 2008. https://apps.who.int/iris/handle/10665/69746 | Yes |  |
| 4 | World Bank. Trauma incidence and emergency medical services in Malawi. World Bank; 2021. https://documents1.worldbank.org/curated/en/176181642602064254/pdf/Trauma-Incidence-and-Emergency-Medical-Services-in-Malawi.pdf | Yes |  |
| 5 | World Health Organization, Malawi Country Office. WHO Malawi country office comprehensive annual report 2024. WHO Regional Office for Africa; 2025. https://www.afro.who.int/sites/default/files/2025-10/WHO%20MALAWI%202024%20ANNUAL%20REPORT_0.pdf | Yes |  |
| 6 | Republic of Malawi, Ministry of Finance and Economic Affairs. Annual economic report 2025. Government of Malawi; 2026. https://finance.gov.mw/documents/uploads/2026-01/Annual%20Economic%20Report%202025.pdf | Yes |  |
| 7 | Republic of Malawi, Ministry of Finance and Economic Affairs. Regional climate resilience program for eastern and southern Africa: Series of projects 2 (P181308), Malawi. Government of Malawi; 2023. https://documents1.worldbank.org/curated/en/099110823152512414/pdf/P1813080dc4197024091eb0c3960c185fba.pdf | Yes |  |
| 8 | International Monetary Fund. Malawi: Economic development document (IMF Country Report No. 17/184). International Monetary Fund; 2017. https://www.imf.org/-/media/files/publications/cr/2017/cr17184.pdf | Yes |  |
| 9 | Republic of Malawi, Ministry of Finance and Economic Affairs. Budget statements. Government of Malawi; n.d. https://www.finance.gov.mw/index.php/resources/budget-statements |  | Yes- No trauma/burn-specific financing data. |
| 10 | Republic of Malawi, Ministry of Finance and Economic Affairs. The 2025/2026 budget policy statement. Government of Malawi; 2025. https://www.finance.gov.mw/documents/uploads/2026-01/Budget%20Policy%20Statement%202025-26_0.pdf | Yes |  |
| 11 | Republic of Malawi, Ministry of Finance and Economic Affairs. Draft 2025/26 financial statement. Government of Malawi; 2025. https://www.finance.gov.mw/documents/uploads/2026-01/Financial%20Statement%202025-26.pdf | Yes |  |
| 12 | Health Sector Strategic Plan II (2017–2022). Government of the Republic of Malawi, Ministry of Health; 2017. PDF available via WHO planning repository: https://extranet.who.int/countryplanningcycles/planning-cycle-files/malawi-malawi-health-sector-strategic-plan-ii-2017-2022 |  | Yes – No specific data on trauma or burn financing. |
| 13 | United Nations Country Team Malawi. UN Malawi Country Results Report 2022. United Nations; 2023. https://malawi.un.org/sites/default/files/2023-08/02_UN_COUNTRY_RESULTS_REPORT.pdf |  | Yes – Does not address trauma/burn care financing. |
| 14 | African Development Bank. Malawi Country Strategy Paper 2023–2028. AfDB; 2023. https://www.afdb.org/en/documents/malawi-country-strategy-paper-2023-2028 |  | Yes – Focuses on macro-development; no trauma/burn funding details. |

**Table S6. Quality appraisal outcomes of the appraised qualitative study using the Mixed Methods Appraisal Tool**

| **No.** | **Author (Year)** | **Study Design** | **Responses to the Appraisal Questions for Qualitative Studies** | | | | | | |
| --- | --- | --- | --- | --- | --- | --- | --- | --- | --- |
|  |  |  | Are there clear research questions? | Do the collected data allow to address the research questions? | Is the qualitative approach appropriate to answer the research question? | Are the qualitative data collection methods adequate to address the research question? | Are the findings adequately derived from the data? | Is the interpretation of results sufficiently substantiated by data? | Is there coherence between qualitative data sources, collection, analysis and interpretation? |
| 1 | Nil. | Nil. | Nil. | Nil. | Nil. | Nil. | Nil. | Nil. | Nil. |

**Table S7. Quality appraisal outcomes of the appraised quantitative randomized studies using the Mixed Methods Appraisal Tool**

| **No.** | **Author (Year)** | **Study Design** | **Responses to the Appraisal Questions for Quantitative Randomized Studies** | | | | | | |
| --- | --- | --- | --- | --- | --- | --- | --- | --- | --- |
|  |  |  | Are there clear research questions? | Do the collected data allow to address the research questions? | Is randomization appropriately performed? | Are the groups comparable at baseline? | Are there complete outcome data? | Are outcome assessors blinded to the intervention provided? | Did the participants adhere to the assigned intervention? |
| 1 | Nil | Nil | Nil | Nil | Nil | Nil | Nil | Nil | Nil |

**Table S8. Quality appraisal outcomes of the appraised quantitative non-randomized studies using the Mixed Methods Appraisal Tool**

| **No.** | **Author (Year)** | **Study Design** | **Responses to the Appraisal Questions for Quantitative Non-randomized Studies** | | | | | | |
| --- | --- | --- | --- | --- | --- | --- | --- | --- | --- |
|  |  |  | Are there clear research questions? | Do the collected data allow to address the research questions? | Are the participants representative of the largest population? | Are measurements appropriate regarding both the outcome and intervention (or exposure)? | Are there complete outcome data? | Are the confounders accounted for in the design and analysis? | During the study period, is the intervention administered (or exposure occurred) as intended? |
| 1 | Gallaher et al. (2015) | Cohort study (Retrospective) | Yes | Yes | Yes | Yes | I can’t tell | Yes | Yes |
| 2 | Purcell et al. (2020) | Cohort study (Retrospective) | Yes | Yes | I can’t tell | Yes | I can’t tell | Yes | Yes |
| 3 | Twea et al. (2024) | Cross-sectional analytical study | Yes | Yes | I can’t tell | Yes | Yes | Yes | Yes |
| 4 | Mody et al. (2023) | Cross-sectional analytical study | Yes | Yes | No | Yes | Yes | Yes | Yes |

**Table S9. Quality appraisal outcomes of the appraised quantitative descriptive study using the Mixed Methods Appraisal Tool**

| **No.** | **Author (Year)** | **Study Design** | **Responses to the Appraisal Questions for Quantitative Descriptive Studies** | | | | | | |
| --- | --- | --- | --- | --- | --- | --- | --- | --- | --- |
|  |  |  | Are there clear research questions? | Do the collected data allow to address the research questions? | Is the sampling strategy relevant to address the research question? | Is the sample representative of the target population? | Are the measurements appropriate? | Is the risk of nonresponse bias low? | Is the statistical analysis appropriate to answer the research question? |
| 1 | Whitaker et al. (2024a) | Cross-sectional descriptive study | Yes | Yes | Yes | Yes | Yes | Yes | Yes |
| 2 | Whitaker et al. (2024b) | Cross-sectional descriptive study | Yes | Yes | Yes | Yes | Yes | Yes | Yes |

**Table S10: Quality appraisal outcomes of the appraised grey literature using the AAOCDS Tool**

| **Checklist** | **Interburns (2019)** | **Trent-Gurbuz (2018)** | **Mock *et al.* (2008)** | **World Bank (2021)** | **WHO (2024)** | **Republic of Malawi, Annual Economic Report (2025)** | **Republic of Malawi, Ministry of Finance and Economic Affairs (2023)** | **International Monetary Fund (2017)** | **Republic of Malawi, Ministry of Finance and Economic Affairs (2025)** | **Republic of Malawi, Ministry of Finance and Economic Affairs**  **(2025)** |
| --- | --- | --- | --- | --- | --- | --- | --- | --- | --- | --- |
| **Authority** |  |  |  |  |  |  |  |  |  |  |
| Associated with a reputable organisation? | Yes | Yes | Yes | Yes | Yes | Yes | Yes | Yes | Yes | Yes |
| Professional qualifications or considerable experience? | Yes | Yes | Yes | Yes | Yes | Yes | Yes | Yes | Yes | Yes |
| Produced/published other work (grey/black) in the field? | Yes | Yes | Yes | Yes | Yes | Yes | Yes | Yes | Yes | Yes |
| Recognised expert, identified in other sources? | Yes | Yes | Yes | Yes | Yes | Yes | Yes | Yes | Yes | Yes |
| Cited by others? | Yes | Not stated | Not stated | Yes | Yes | Yes | Yes | Yes | Yes | Yes |
| Higher degree student under “expert” supervision? | Not applicable | Not applicable | Not applicable | Not applicable | Not applicable | Not applicable | Not applicable | Not applicable | Not applicable | Not applicable |
| Is the organisation reputable? | Yes | Yes | Yes | Yes | Yes | Yes | Yes | Yes | Yes | Yes |
| Is the organisation an authority in the field? | Yes | Yes | Yes | Yes | Yes | Yes | Yes | Yes | Yes | Yes |
| Does the item have a detailed reference list or bibliography? | No | Yes | Yes | Yes | No | No | No | No | No | No |
| **Accuracy** |  |  |  |  |  |  |  |  |  |  |
| Does the item have a clearly stated aim or brief? | Yes | Yes | Yes | Yes | Yes | Yes | Yes | Yes | Yes | Yes |
| If so, is this met? | Yes | Yes | Yes | Yes | Yes | Yes | Yes | Yes | Yes | Yes |
| Does it have a stated methodology? | Yes | No | Yes | Yes | Yes | Yes | Yes | Yes | Yes | Yes |
| If so, is it adhered to? | Yes | Not applicable | Yes | Yes | Yes | Yes | Yes | Yes | Yes | Yes |
| Has it been peer-reviewed? | No | No | No | No | No | Not stated | Not stated | Not stated | No | No |
| Has it been edited by a reputable authority? | Yes | Yes | Yes | Yes | Yes | Yes | Yes | Yes | Yes | Yes |
| Supported by authoritative, documented references or credible sources? | Yes | No | Yes | Yes | Yes | Yes | Yes | Yes | Yes | Yes |
| Is it representative of work in the field? | Yes | Yes | Yes | Yes | Yes | Yes | Yes | Yes | Yes | Yes |
| If No, is it a valid counterbalance? | Not applicable | Not applicable | Not applicable | Not applicable | Not applicable | Not applicable | Not applicable | Not applicable | Yes | Yes |
| Is any data collection explicit and appropriate for the research? | Not stated | No | Yes | Yes | Yes | Yes | Yes | Yes | Yes | Yes |
| If item is secondary material refer to the original. Is it an accurate, unbiased interpretation or analysis? | Not applicable | Yes | Yes | Not applicable | Yes | Yes | Yes | Yes | Yes | Yes |
| The original, is it an accurate, unbiased interpretation or analysis? | No | No | Yes | Yes | Yes | Yes | Yes | Yes | Yes | Yes |
| **Coverage** |  |  |  |  |  |  |  |  |  |  |
| Are any limits clearly stated? | Yes | Yes | Yes | Yes | Yes | Yes | Yes | Yes | Yes | Yes |
| **Objectivity** |  |  |  |  |  |  |  |  |  |  |
| Opinion, expert or otherwise, is still opinion: is the author’s standpoint clear? | Yes | Yes | Yes | Yes | Yes | Yes | Yes | Yes | Yes | Yes |
| Does the work seem to be balanced in presentation? | No | No | Yes | Yes | Yes | Yes | Yes | Yes | Yes | Yes |
| **Date** |  |  |  |  |  |  |  |  |  |  |
| Does the item have a clearly stated date related to content? | No stated | Yes | Yes | Yes | Yes | Not stated | Yes | Yes | Yes | Yes |
| If no date is given, but can be closely ascertained, is there a valid reason for its absence? | Not applicable | Not applicable | Not applicable | Not applicable | Not applicable | No | Not applicable | Not applicable | Not applicable | Not applicable |
| Check the bibliography: have key contemporary material been included? | Not stated | Not stated | Yes | Yes | Not stated | Not stated | Not stated | Not stated | Yes | Yes |
| **Significance** |  |  |  |  |  |  |  |  |  |  |
| Is the item meaningful? (this incorporates feasibility, utility and relevance) | Yes | Yes | Yes | Yes | Yes | Yes | Yes | Yes | Yes | Yes |
| Does it add context? | Yes | Yes | Yes | Yes | Yes | Yes | Yes | Yes | Yes | Yes |
| Does it enrich or add something unique to the research? | Yes | Yes | Yes | Yes | Yes | Yes | Yes | Yes | Yes | Yes |
| Does it strengthen or refute a current position? | Yes | Yes | Yes | Yes | Yes | Yes | Yes | Yes | Yes | Yes |
| Would the research area be lesser without it? | Yes | Yes | Yes | Yes | Yes | Yes | Yes | Yes | Yes | Yes |
| Is it integral, representative, typical? | Yes | Yes | Yes | Yes | Yes | Yes | Yes | Yes | Yes | Yes |
| Does it have impact? | Yes | Yes | Yes | Yes | Yes | Yes | Yes | Yes | Yes | Yes |
